# Analysis of the cell wall binding domain in bacteriocin-like lysin LysL from *Lactococcus lactis* LAC460

**DOI:** 10.1007/s00203-024-04066-5

**Published:** 2024-07-02

**Authors:** Samira Mokhtari, Yanru Li, Per E. J. Saris, Timo M. Takala

**Affiliations:** Department of Microbiology, University of Helsinki, P.O. Box 56, 00014 Helsinki, Finland

**Keywords:** *Lactococcus lactis*, Prophage lysin, Green fluorescent protein, Cell wall binding domain

## Abstract

Wild-type *Lactococcus lactis* strain LAC460 secretes prophage-encoded bacteriocin-like lysin LysL, which kills some *Lactococcus* strains, but has no lytic effect on the producer. LysL carries two N-terminal enzymatic active domains (EAD), and an unknown C-terminus without homology to known domains. This study aimed to determine whether the C-terminus of LysL carries a cell wall binding domain (CBD) for target specificity of LysL. The C-terminal putative CBD region of LysL was fused with His-tagged green fluorescent protein (HGFPuv). The *HGFPuv_CBDlysL* gene fusion was ligated into the pASG-IBA4 vector, and introduced into *Escherichia coli*. The fusion protein was produced and purified with affinity chromatography. To analyse the binding of HGFPuv_CBDLysL to *Lactococcus* cells, the protein was mixed with LysL-sensitive and LysL-resistant strains, including the LysL-producer LAC460, and the fluorescence of the cells was analysed. As seen in fluorescence microscope, HGFPuv_CBDLysL decorated the cell surface of LysL-sensitive *L. cremoris* MG1614 with green fluorescence, whereas the resistant *L. lactis* strains LM0230 and LAC460 remained unfluorescent. The fluorescence plate reader confirmed the microscopy results detecting fluorescence only from four tested LysL-sensitive strains but not from 11 tested LysL-resistant strains. Specific binding of HGFPuv_CBDLysL onto the LysL-sensitive cells but not onto the LysL-resistant strains indicates that the C-terminus of LysL contains specific CBD. In conclusion, this report presents experimental evidence of the presence of a CBD in a lactococcal phage lysin. Moreover, the inability of HGFPuv_CBDLysL to bind to the LysL producer LAC460 may partly explain the host’s resistance to its own prophage lysin.

**Supplementary Information:**

The online version contains supplementary material available at 10.1007/s00203-024-04066-5.

## Introduction

*Lactococcus lactis* is highly valued in the dairy industry for its dual role as a starter in many different fermented products and in aiding food preservation (Lortal and Chapot-Chartier 2005). All lactococcal genomes harbor prophages; genes encoding lysins are present in many of these prophage regions. These lytic enzymes can degrade peptidoglycan in the cell wall, ultimately resulting in cell lysis and cell death (Oliveira et al. 2018).

Phage lysins are generally divided into two groups, namely endolysins and virion-associated lysins (VALs; Abdelrahman et al. 2021). VALs typically exhibit dual functions as both phage structural components and lytic enzymes. VALs are found in different parts of phages, including tails, where they locally degrade the peptidoglycan of target bacteria during phage infection, without lysing the cell. This enzymatic activity facilitates entry of the phage’s genetic material into the host cell (Oliveira et al. 2018). Endolysins, on the other hand, have a role at the end of the life cycle when newly formed phages are released from the host cells. These enzymes work by degrading the peptidoglycan layer of the infected cell from inside, facilitating phage release (Oechslin et al. 2022).

Endolysins targeting Gram-positive bacteria typically consist of one or two enzymatic active domains (EAD) along with a cell wall binding domain (CBD). VALs are typically composed of one or two EADs and are believed to lack a CBD (Chandran et al. 2022). The EAD of an endolysin is usually located in the N-terminus of the lysin. The enzymatic activity is either N-acetylmuramidase or endo-β-*N*-acetylglucosaminidase, which cleave the sugar backbone of the peptidoglycan, endopeptidase cleaving the peptide segment of the cell wall, or *N*-acetylmuramyl-l-alanine amidase, hydrolyzing the amide bonds connecting the sugar chain to the peptide (Broendum et al. 2018; Loessner 2005). The activity of the lysin also requires binding to the cell wall; phage endolysins do not work effectively if they are not bound to the cell wall (Loessner et al. 2002). The CBD is usually located in the C-terminus, which is responsible for the enzyme’s selective binding to the cell wall, ultimately defining the specificity of the lysin (Broendum et al. 2018). Several CBDs have been recognized, with LysM standing out as one of the most familiar. LysM is found in motifs and specifically binds to *N*-acetylglucosamine residues of peptidoglycan (Raya-Tonetti et al. 2021). Another well-established CBD is SH3, which exists in five subgroups and binds to peptidoglycan and facilitates protein–protein interactions (Desvaux et al. 2018). Among *Lactococcus* phage lysins, CBD types CHW, LysM, PG-1, SH3-5, and SH3b have been identified by sequence analyses (Oliveira et al. 2013). In addition, many lactococcal phage lysins carry uncharacterized regions, which may serve as binding domains. Of these putative anchoring domains, only a few have been experimentally demonstrated to function as CBDs (Plavec et al 2019).

**Fig. 1:**
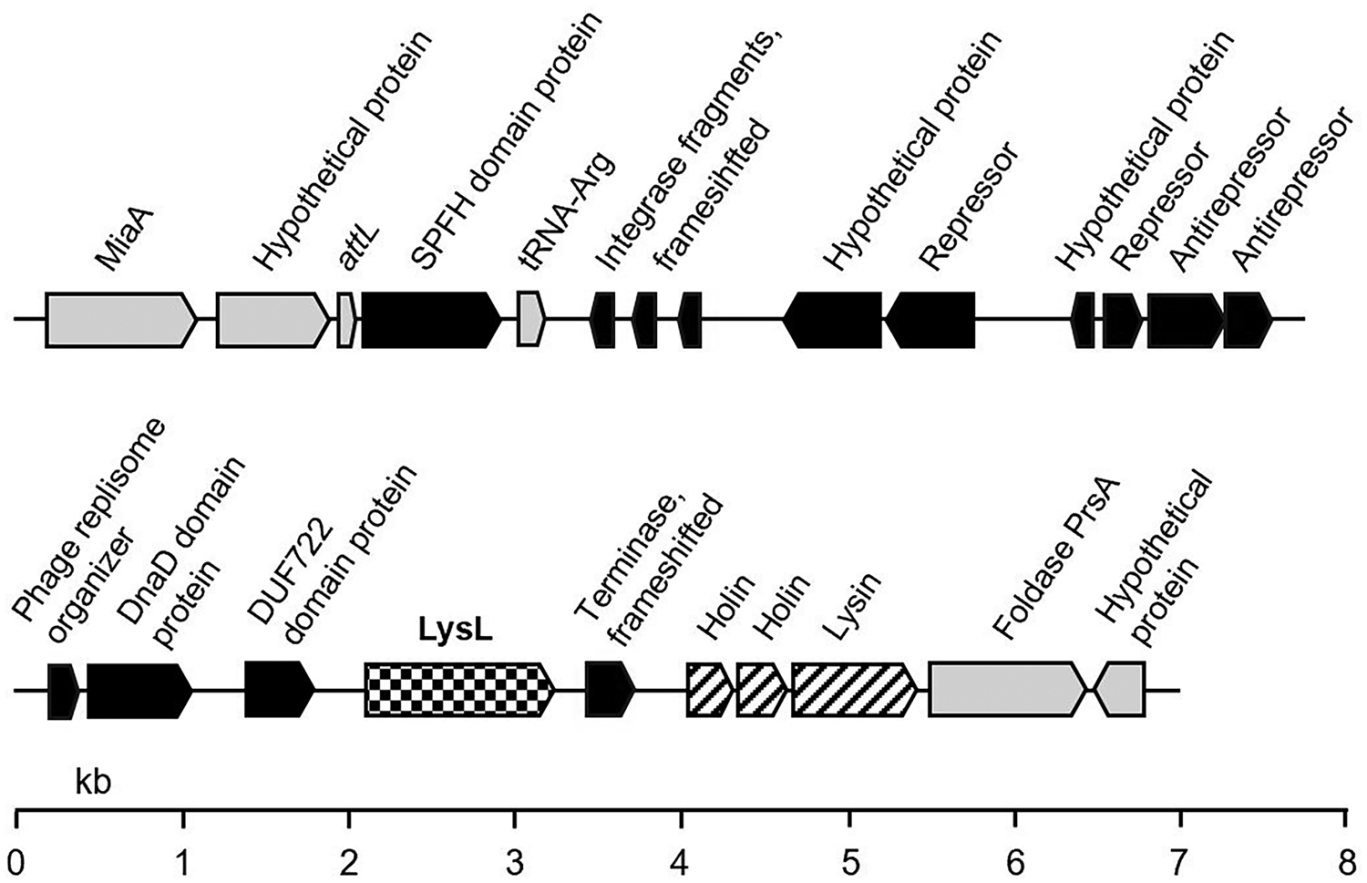
Location of the *lysL* gene in the defective prophage PLl460-1 and the flanking bacterial genes in *L. lactis* LAC460 chromosome. The encoded predicted proteins are shown above the genes. Bacterial genes are in light grey, putative phage genes in black, *lysL* gene in checker board pattern, and the phage lysis cassette genes, including two holins and a putative endolysin, in diagonal stripes. Several phage genes are non-functional, partial, or frameshifted pseudogenes

In our previous work, we reported a bacteriocin-like lysin LysL (accession number UCS91464.1), produced by *L*. *lactis* strain LAC460 isolated from spontaneously fermented idli batter (Takala et al. 2023). LysL is a 385-aa lysin originating from a defective prophage PLl460-1 in the chromosome of *L. lactis* LAC460. Figure 1 shows the prediction of the genes in PLl460-1, and the location of the *lysL* gene in the prophage, according to phage search tool PHASTEST, open reading frame finder ORFfinder, and protein sequence comparison tool BlastP (https://phastest.ca, https://www.ncbi.nlm.nih.gov/orffinder and https://blast.ncbi.nlm.nih.gov/Blast.cgi, respectively). LysL is heat-sensitive, is produced and secreted in late log-phase and early stationary phase, and does not break down the host’s cell wall but kills other *Lactococcus* strains like a bacteriocin. According to the sequence and conserved domain homologies, LysL possesses EADs of lysozyme and peptidase M23 (approximately aa residues 7-270). However, the function of the C-terminus of about 115 aa is unknown, as no homology to known domains has been detected.

The aim of this study was to investigate the target specificity of LysL by determining the presence or absence of a CBD in its unknown C-terminus. It was found that the CBDLysL binds only to LysL sensitive *Lactococcus* sp. strains, but not to LysL resistant strains, which may explain the host’s resistance to its own phage lysin.

## Materials and methods

### Bacterial strains, plasmids, and culture conditions

Bacterial strains and plasmids used in this study are shown in Table 1, except the *Lactococcus* sp. strains used only in cell wall polysaccharide (CWPS) genotyping are shown in Table 2 in Results section.* E. coli* strains were grown in LB medium (1% tryptone, 0.5% yeast extract, 1% NaCl) with shaking (200 rpm) at 37 °C. Ampicillin (150 μg ml^−1^) was used for selecting transformants. *Lactococcus* strains were grown in M17 (Oxoid Ltd. Basingstoke, UK) broth supplemented with 0.5% (w/v) glucose (M17G) at 30 °C. Chloramphenicol (10 μg ml^−1^) was added to the growth media for *Lactococcus cremoris* LAC275. Solid media were prepared by adding 1.5% agar to the broth media.

**Table 1:** Bacterial strains and plasmids used in this study

Bacterial strain/plasmid	Relevant properties	References/source
*Escherichia coli* DH5α	Transformation and gene expression host	Hanahan (1985)
*E. coli* ECO809	*E. coli* TG1 carrying plasmid pLEB798, a derivative of the pASG-IBA4 vector	Sorokina (2015)
*E. coli* ECO854	*CBDlysL* clone; DH5α carrying plasmid pLEB836 with tetracycline-inducible *HGFPuv_CBDlysL* expression	This study
*Lactococcus cremoris* LAC275	Source of *GFPuv* gene; carries *GFPuv* expression plasmid; Cam^R^	Hakovirta et al. (2006)
*L. cremoris* MG1614	Indicator strain, sensitive to LysL	Gasson (1983)
*L. lactis* LM0230	Indicator strain, resistant to LysL	Efstathiou and Mckay (1977)
*L. lactis* LAC460	Wild-type LysL producer, resistant to LysL	Takala et al. (2023)
pASG-IBA4	*E. coli* expression vector carrying tetracycline-inducible promoter and tet repressor gene; *ampR*	IBA Lifesciences GmbH, Göttingen, Germany

**Table 2:** CBDLysL binding, LysL sensitivity, and CWPS genotype of different *Lactococcus* strains

Strain	CWPS genotype	LysL sensitivity	CBDLysL binding	Source/References of the strain
*L. lactis* ATCC 11454	A	–	–	Steen et al. (1991)
*L. lactis* 279	A	–	–	Prof. Ömer Simsek, Pamukkale University, Turkey
*L. lactis* 91/1	A	–	–	Valio Ltd., Helsinki, Finland
*L. lactis* N8	A	–	–	Wan et al. (2021)
*L. cremoris* SSC181	B	–	–	Valio Ltd
*L. lactis* IL1403	B	–	–	Chopin et al. (1984)
*L. cremoris* MG1614	C	+	+	Gasson (1983)
*L. lactis* L71	C	+	+	Colombo (2016), Takala et al. (2023)
*L. lactis* SSL110	C	–	–	Valio Ltd
*L. lactis* ATCC 19435	Other/unknown	+	+	ATCC, Manassas, VA, USA
*L. lactis* ATCC 7962	Other/unknown	–	–	ATCC
*L. lactis* FA73	Other/unknown	–	–	Colombo (2016), Takala et al. (2023)
*L. lactis* LAC460	Other/unknown	–	–	Takala et al. (2023)
*L. lactis* LM0230	Other/unknown	–	–	Efstathiou and McKay (1977)
*L. lactis* NCDO1404	Other/unknown	+	+	NCDO/NCIMB, Aberdeen, UK

+ the strain is sensitive to LysL and binds CBDLysL, – the strain is resistant to LysL and does not bind CBDLysL

### DNA techniques

The PCR primers used in this study are presented in the Supplementary Table 1. Phusion High-Fidelity DNA polymerase (Thermo Scientific, Waltham, MA, USA) was used in PCR according to the instructions of the producer. GeneJET PCR purification kit and GeneJET Gel Purification Kit (Thermo Scientific) were used for DNA purification of PCR products and from agarose gel according to the manufacturer’s instructions, respectively. CWPS genotyping of *Lactococcus* sp. strains was done by multiplex PCR according to Mahony et al. (2013), except the used control primers targeted bacterial 16S rRNA gene (Edwards et al. 1989). Plasmid DNA was isolated from the recombinant cultures with a GeneJET Plasmid Miniprep Kit (Thermo Scientific) as instructed by the manufacturer.

### Construction of *HGFPuv_CBDlysL* expression plasmid and transformation

According to BLAST matches, the EADs in the *lysL* gene constitute the first 810 bp of the entire length of 1158 bp. Therefore, the rest of the gene was considered as potentially encoding for the putative CBD region, and the last 360 nucleotides were amplified by using *L. lactis* LAC460 cells as a template with LysL F OE GFP and LysL R primers. The UV light-excited green fluorescent (GFPuv) gene lacking the stop codon but including hexahistidine (*6* × *His-tag*) codons was amplified by PCR using *L. cremoris* LAC275 cells as template with the His-GFP F and GFP R OE L primers. As the actual Overlap Extension PCR for constructing the *HGFPuv*_*CBDlysL* fusion only produced strong bands of wrong size, the *HGFPuv* and *CBDlysL* fragments were joined together by polymerization without primers using standard PCR program. The insert *HGFPuv_CBDlysL* was phosphorylated by T4 polynucleotide kinase (Thermo Scientific) according to manufacturer’s instructions at 37 °C for 30 min, heat inactivated at 65 °C for 20 min, and purified with PCR purification kit. The vector pASG-IBA4 was amplified without its *ompA* signal sequence by PCR using *E. coli* ECO809 cells carrying a derivative of pASG-IBA4 plasmid as a template with pASG-IBA4 F and IBA noSS R primers, followed by purifying the vector fragment with a PCR purification kit. The insert and the vector fragments were ligated using T4 DNA ligase (Thermo Scientific) overnight at room temperature, as instructed by the manufacturer.

The ligation mixture was then electroporated into *E. coli* DH5α (Zabarovsky and Winberg 1990). Cells were spread onto selective plates (LB Amp^150^) and incubated at 37 °C overnight. Obtained transformant colonies were screened by PCR with His-GFP F and LysL R primers. The plasmid was then extracted from the correct clones and sequenced to confirm the correct construct. The recombinant *E. coli* DH5α carrying the correct pASG-IBA4-*HGFPuv_CBDlysL* plasmid pLEB836 was stored as *E. coli* ECO854.

### Western blot and purification of HGFPuv_CBDLysL

HGFPuv_CBDLysL was purified with affinity chromatography by an outsourced protein purification service (Protein Service core facility of the Tampere University). Briefly, 200 ml of *E. coli* ECO854 culture in LB Amp^150^ was induced with 0.5 μg ml^−1^ tetracycline after OD_600_ reached about 0.5. The cells were harvested by centrifugation (5000×*g*, 10 min) after 20 h of continued incubation. The cell pellet was resuspended in 50 ml of PBS (pH 7.2) and treated four times with an Emulsiflex C3 high-pressure homogenizer (Avestin Inc., Ottawa, Canada). The content was then centrifuged (30,000×*g*, 4 °C, 20 min) and the supernatant was used as a cell lysate sample.

Western blotting was performed essentially as described by Towbin et al. (1979). SDS-PAGE of the cell lysate proteins was carried out as described by Laemmli (1970) excluding the staining and destaining steps. Briefly, the cell lysate was mixed in a 5:1 ratio with Tris–Glycine SDS-PAGE loading buffer (Bio-Rad Laboratories, Hercules, CA, USA) and heated at 95°C for 5 min. Then, 5 µl of the sample was loaded into a gradient SDS-PAGE gel (4–20%, Bio-Rad Laboratories) and electrophoresed at 200 V for 30 min at room temperature. Electrotransfer of the protein bands from SDS gel to a cellulose nitrate membrane was done under the voltage 120 V at 4 °C for 40 min. The membrane was then exposed to blocking solution (10% (w/v) bovine serum albumin) for 1 h with mild agitation followed by washing three times with TBS-T buffer (150 mM NaCl, 25 mM Tris, 0.1% Tween 20, pH 7.5) for 5 min. The membrane was then probed with primary mouse anti-His.H8 diluted 1:10,000 in 5% milk-TBST (Thermo Scientific) at 4 °C overnight with mild agitation followed by washing three times with TBST buffer for 5 min. The membrane was then incubated with horse anti-mouse IgG(H + L) Peroxidase (Vector Laboratories, Newark, CA, USA) at 1:20 000 dilution in TBST for 1 h at room temperature. Afterward, the membrane was washed three times with TBST buffer for 5 min each time. Protein bands were visualized using the WesternBright ECL HRP substrate (Advansta, San Jose, CA, USA) following the manufacturer’s instructions. Imaging was performed using the ChemiDoc MP Imaging System (Bio-Rad Laboratories).

For purification of HGFPuv_CBDLysL, the cell lysate was obtained from 900 ml of tetracycline-induced culture of *E. coli* ECO854 as described above. 10 mM imidazole was added to the pellet lysate sample to prevent nonspecific binding, then the sample was bound to HisPur™ Ni–NTA agarose (Thermo Scientific) in a batch mode at 4 °C for 1 h. The bound protein was washed with 10 column volumes with Wash Buffer (PBS, 250 mM NaCl, 50 mM imidazole, pH 7.2) before stepwise elution with 5 column volumes of Elution Buffer (20 mM Tris–HCl, 500 mM NaCl, 250 mM imidazole, pH 7.5). NanoDrop One (Thermo Scientific) was used for measuring protein concentration of the samples at A_280_.

### Cell wall binding assays

Binding of HGFPuv_CBDLysL onto *Lactococcus* cells was determined by the binding assay described previously (Loessner et al. 2002). The indicator strains (Table 2) were cultured (10 ml) overnight, harvested by centrifugation (5000×*g*, 10 min), and resuspended in the same volume of PBS-T buffer (pH 7.4, 0.1% Tween 80). 600 µl of cell suspensions were mixed with 5 µl of the purified HGFPuv_CBDLysL (1.73 mg/ml) and incubated at room temperature for 15 min. Cells were pelleted (5000×*g*, 5 min), washed with 1 ml of PBS-T, resuspended in 600 µl of PBS-T, and applied to a microplate. Fluorescence was measured with Infinite M200 plate reader (Tecan, Männedorf, Switzerland) with excitation at 395 nm and emission at 508 nm. Part of the cell suspensions of strains *L. cremoris* MG1614, *L. lactis* LM0230 and *L. lactis* LAC460 were also used for visualization with a Zeiss Axioscope 2 Plus fluorescence microscope using Axiocam 305 color camera and Carl Zeiss microscope software 2.6 pre (Carl Zeiss Microscopy, Jena, Germany) with a WTGFP filter (502 nm emission).

## Results 

### Cloning and expression of HGFPuv_CBDLysL in *E. coli*

With the purpose of producing GFPuv-fused CBD of LysL, *HGFPuv_CBDLysL* was cloned in *E. coli* DH5α. When exposed to UV light, the cell pellet of the tetracycline-induced *E. coli* ECO854 culture showed green fluorescence, whereas the parallel non-induced cell pellet did not (result not shown). This indicates that the cloning and expression of *HGFPuv_CBDLysL* in *E. coli* was successful and that the HGFPuv in the fusion protein is functional.

### Western blot and purification of HGFPuv_CBDLysL

**Fig. 2:**
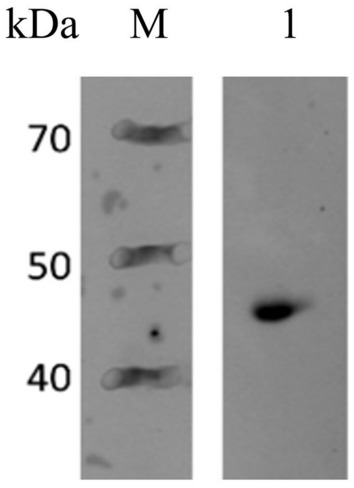
Western blot of the tetracyclin-induced cell lysate of *E. coli* ECO854. Lane 1, HGFPuv_CBDLysL; M, PageRuler Broad Range Protein Ladder (Thermo Scientific)

Western blotting was performed prior to purification to verify the presence of LysL(CBD) and the N-terminal 6 × His-tag in the fusion protein in cell lysate of *E. coli* ECO854. As the molecular weight of HGFPuv is approximately 28 kDa and CBD_LysL about 14 kDa, the distinct band above 40 kDa on the blotted membrane represents HGFPuv_CBDLysL, recognized by the anti-His antibody (Fig. 2). Purification of HGFPuv_CBDLysL was performed using 6 × His-tag and HisPur™ Ni–NTA chromatography. From the 900 ml of the initial culture of *E. coli* ECO854, the total protein yield was 3.06 mg with the concentration of 1.73 mg/mL.

### Binding of HGFPuv_CBDLysL protein onto *Lactococcus* cells

**Fig. 3:**
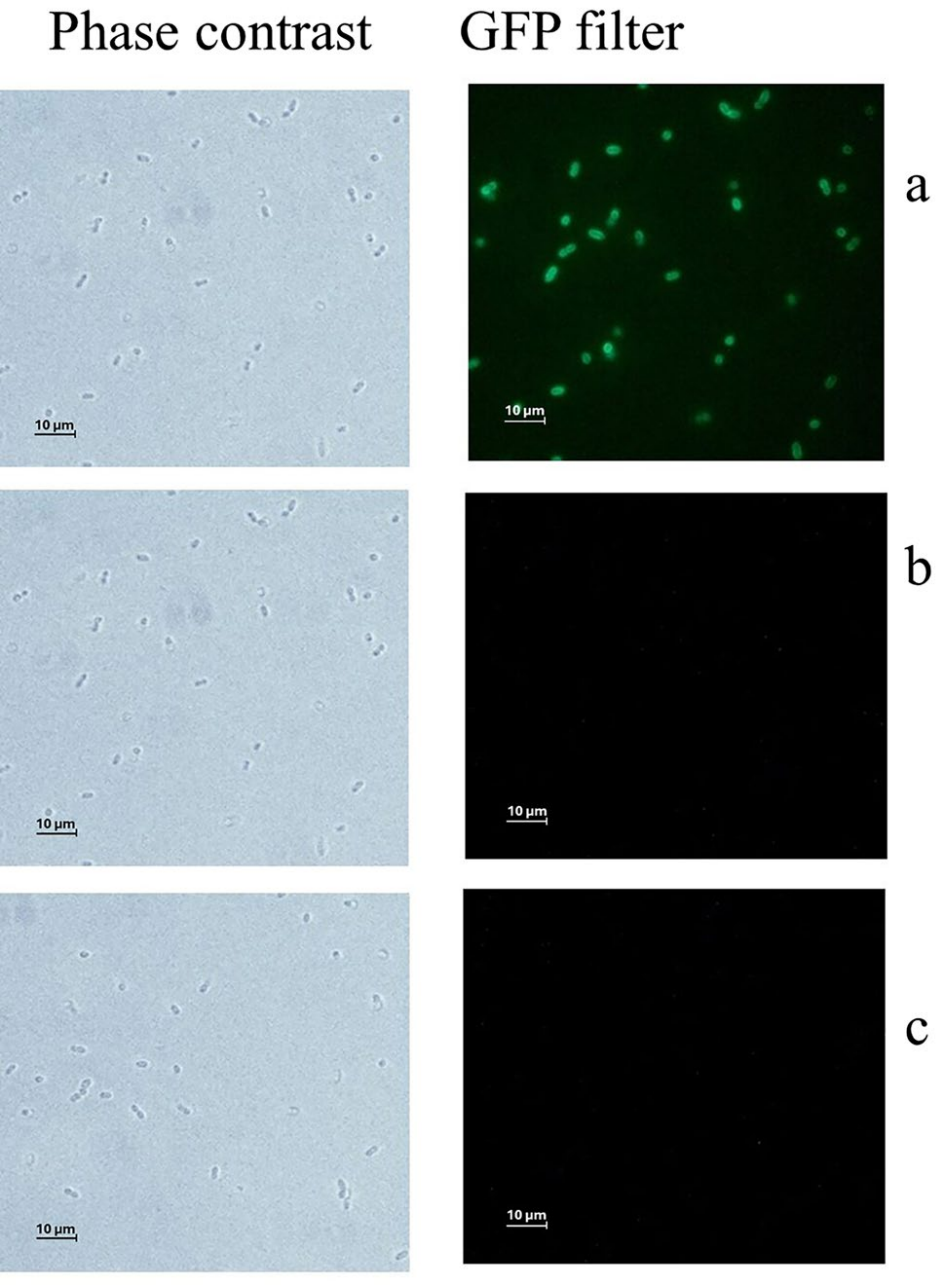
Phase contrast and fluorescence microscopy images of *L. cremoris* MG1614 (**a**), *L. lactis* LM0230 (**b**), and *L. lactis* LAC460 (**c**) cells mixed with HGFPuv_CBDLysL protein. Only *L. cremoris* MG1614 cells were decorated with green fluorescence

The purified HGFPuv_CBDLysL was used to examine the specific binding of CBDLysL to the cell wall of the *Lactococcus* strains. As shown in the microscope images (Fig. 3), green fluorescence was only observed on the *L. cremoris* MG1614 cell surface, demonstrating that CBD of LysL binds to the cell wall of the LysL-sensitive *Lactococcus* strain. No green decoration was observed on the cells of the LysL-resistant *L. lactis* LM0230 or the LysL producer strain *L. lactis* LAC460.

**Fig. 4:**
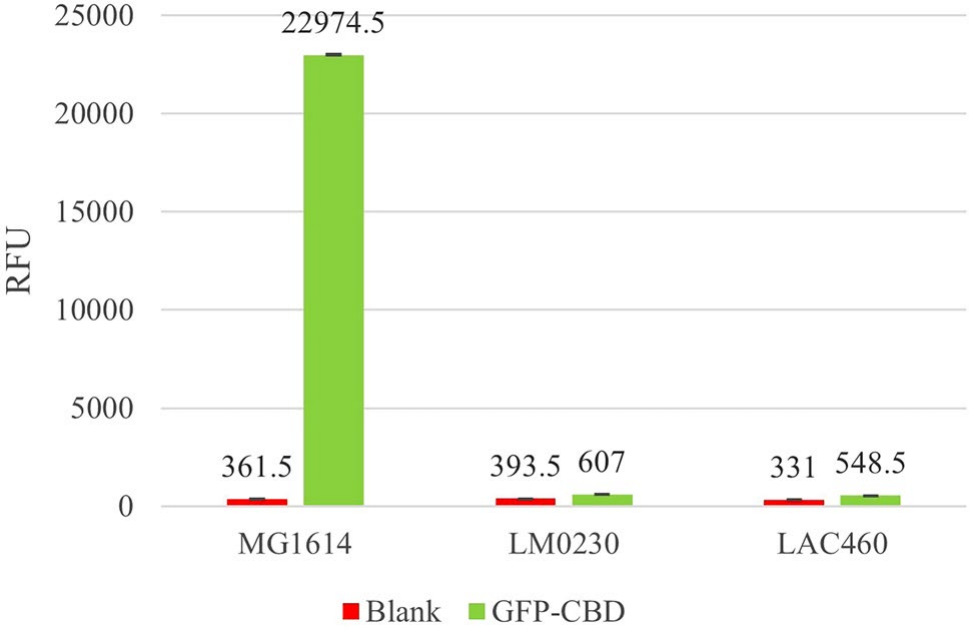
Fluorescence of *Lactococcus* strains mixed with purified HGFPuv_CBDLysL protein measured by a fluorescence plate reader. Blank, cells without added HGFPuv_CBDLysL protein, i.e., background fluorescence from the cells. *RFU* relative fluorescence unit. Error bars represent standard deviation of two duplicates

In addition, binding of the purified HGFP_CBDLysL fusion protein was examined with *Lactococcus* strains representing different CWPS genotypes. The strains’ CWPS genotype was determined by multiplex PCR, LysL sensitivity was tested on indicator plates, and the binding of HGFP_CBDLysL was measured with a fluorescence plate reader. The results are shown in Table 2, and the HGFP_CBDLysL binding to a few selected strains is also shown in Fig. 4. HGFP_CBDLysL only bound to the LysL sensitive strains, and it did not bind to LysL resistant strains. The LysL sensitive strains represented either the CWPS genotype C or an unknown genotype. The LysL resistant strains were found from all CWPS genotypes, showing no clear correlation between the CWPS genotype and the binding of CBDLysL.

### Domain prediction

**Fig. 5:**
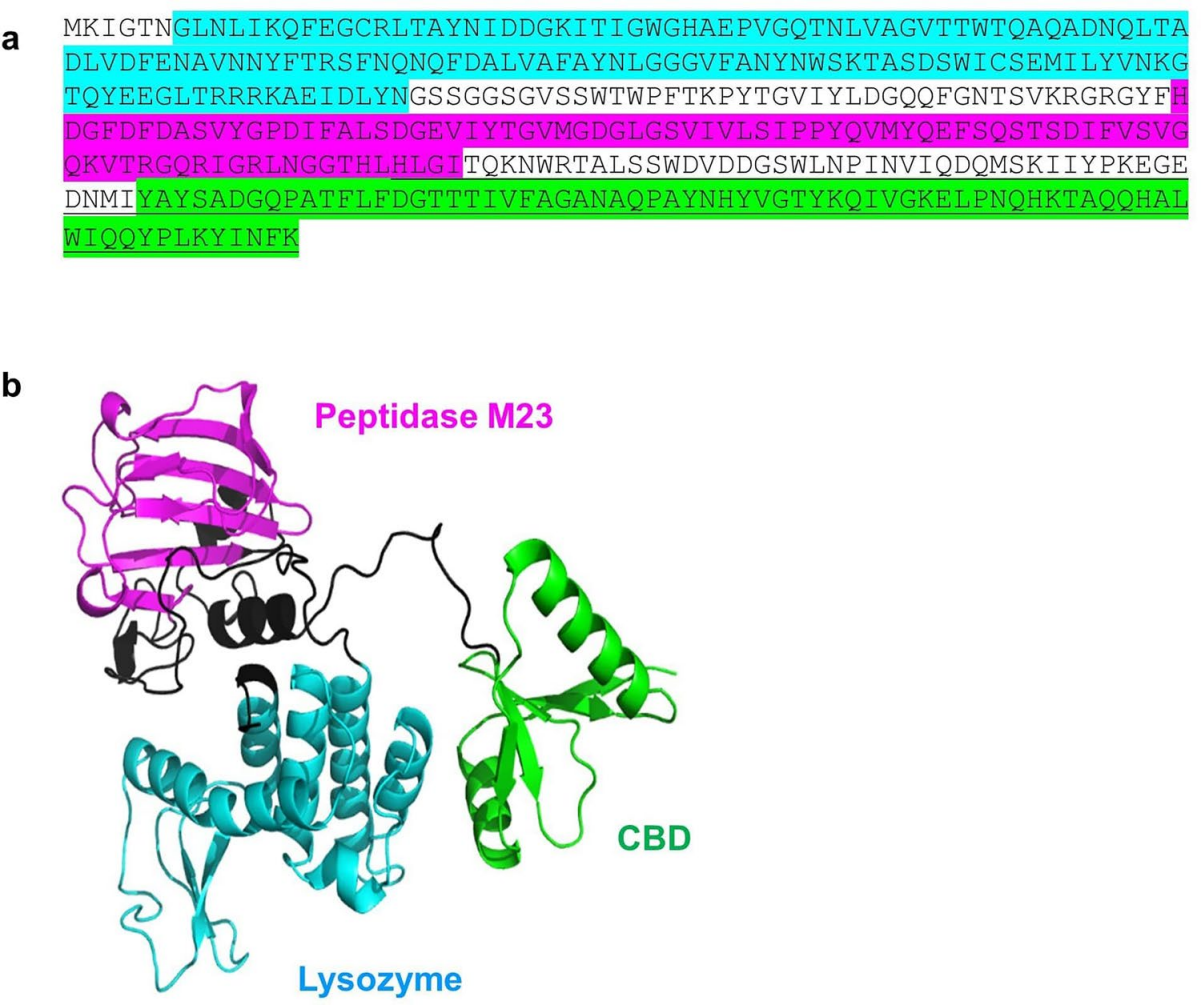
Predicted domains and structure of LysL. **a** Amino acid sequence. The underlined sequence is the 119-aa C-terminal region fused with GFP. **b** structure prediction by Alphafold2. The three distinct domains of lysozyme, peptidase, and cell binding domain are marked in both sequence and 3-D structure with cyan, magenta, and green, respectively. The linkers between the domains are shown in black. Lysozyme and peptidase positions were predicted by NCBI Conserved Domains search tool. The location of CBD is an estimate based on 3-D structure

The results of the binding assay suggest that there is a region that binds the protein onto the cell surface of LysL-sensitive bacteria within the 119 C-terminal amino acids in LysL (Fig. 5a). However, unlike the two N-terminal EADs in LysL, lysozyme and peptidase, the NCBI Conserved Domain search tool did not recognize any structural domain in the C-terminus of LysL. Therefore, we sought to elucidate the three-dimensional structure of LysL by using Alphafold2. Three distinct domains in the 3-D structure of LysL were revealed (Fig. 5b). The two N-terminal structures are likely the lysozyme and peptidase domains predicted by the Conserved Domains search. The third separate structure in the unknown C-terminus is presumably the CBD, demonstrating that the cell binding region forms a distinct structural domain in LysL. The precise location and the length of the CBD, the 58 C-terminal residues forming a seemingly independent structure after a long linker, is still only an estimate based on the 3-D prediction.

## Discussion

This study sought to investigate the cell binding domain of the bacteriocin-like lysin LysL from *Lactococcus lactis* LAC460. The CBD was fused with GFPuv and the fusion protein was mixed with different *Lactococcus* cells to detect the possible binding of the CBD onto the cell surface. The HGFPuv_CBDLysL protein bound to LysL-sensitive strains, but not to LysL-resistant strains, including the LysL-producer *L. lactis* LAC460, as observed with fluorescence microscopy and as measured with a plate reader. The results confirmed that the C-terminus of LysL carries a CBD with strain-specific binding property that determines the target specificity of the LysL lysin. Based on protein BLAST comparisons, identical or similar (78–79% identity) C-terminal domains seem to be linked with M23 peptidases. Identical CBDs are only found in LysL-like enzymes carrying a lysozyme and peptidase domains, but similar (putative) CBDs are also present in enzymes with only M23 peptidase domain. However, these M23 peptidases show only around 50% aa-sequence identities to the peptidase domain of LysL.

The inability of HGFPuv_CBDLysL to bind to *L. lactis* LAC460 may be the reason why LysL does not lyse the host’s own cell wall. It has been suggested that bacteria domesticate prophages for their own benefit, for example to improve secretion, gene transfer, and defense (Bobay et al. 2014). This work provides an example of domestication of a prophage lysin by *L. lactis* strain. However, the receptor for the CBDLysL and the reason why *L. lactis* LAC460 contains remnants of prophages that do not work against the host, remain to be investigated.

There are not many studies about experimental evidence of the function of lactococcal phage lysin CBDs. Roces et al. (2016) have characterized the LysM domain of the endolysin LysTP712, and Plavec et al (2019) have tested a few putative CBDs, including unchacterized domains, for surface display of proteins on *L. lactis* cells. In addition to phage lysin CBDs, also other cell binding proteins, e.g. lactococcal autolysin AcmA carrying LysM repeats, have been exploited for displaying proteins on the *Lactococcus* cell surface (Zahirović et al. 2022). Endolysin CBDs of some other genera have also been studied. For instance, the binding of the CBDs of *Listeria monocytogenes* and lactobacilli phage endolysins has been studied by the same approach as in this work, namely constructing CBD fusions with fluorescent protein and testing protein binding to different bacteria (Dorosky et al. 2023; Schmelcher et al. 2011). In the aforementioned study by Dorosky et al (2023), a method to differentiate specific lactobacilli from a mixture was developed based on CBD binding.

Studies on the receptors of CBD targets are very limited; only a few ligands are known. Of the known CBD targets, the involvement of wall teichoic acids in the binding of *L. monocytogenes* phage lysins to CBDs have been shown (Eugster and Loessner 2012; Eugster et al. 2011). Research by Mahony et al. in 2013 revealed that the host range of 936-type phages correlates with the cell wall polysaccharide (CWPS) types of the targeted *L. lactis* bacteria. Among the 11 lactococcal 936-type phages studied, most infected strains with MG/SK CWPS (genotype C), others with IL/KF CWPS (genotype B), and a small portion demonstrated infectivity towards both. Even though CWPS has been shown to be a major factor determining phage sensitivity in *Lactococcus* spp. (Ainsworth et al 2014), it seems not to correlate with LysL sensitivity, as both LysL sensitive and resistant strains were found from different CWPS genotype groups (Table 2). Consequently, it seems unlikely that the CBDLysL would use CWPS as the binding target. However, additional investigation is required for a better understanding of the specific target to which CBDLysL binds.

It is not fully certain whether LysL is a VAL or endolysin. VALs are often claimed to lack a CBD, although there is an exception reported where the VAL of *Staphylococcus aureus* phage P68 carries a CBD (Takác and Bläsi 2005). Regarding EADs, M23 peptidase present in LysL is rarely an EAD in endolysins, while being frequently found in VAL catalytic active domains (Oliveira et al. 2018). Otherwise the LysL protein structure resembles a conventional endolysin. However, the location of the *lysL* gene in the genome is not typical for an endolysin gene. Endolysin and holin genes are usually located adjacently in the lysis cassette of prophages (Summer et al. 2007; Shin et al. 2014). However, this is not always the case, as for instance in the ΦC2 prophage of *Clostridioides difficile* the endolysin gene is downstream of holin gene after *abiF* gene, which confers phage resistance to bacteria (Goh et al. 2007). In the case of *lysL* gene, there is no adjacent holin gene, but there are two holin genes about 800 bp downstream of *lysL* (Fig. 1). However, immediately downstream of the holin genes, there is another gene encoding a lysin with an N-terminal amidase as an apparent EAD and with an unknown C-terminus for a putative CBD, making this lysin gene to look like a typical endolysin. Still, some phages carry two endolysins, e.g. *Lactococcus* phage KSY1 and *E. coli* phage swi2 (Chopin et al. 2007; Sui et al. 2021). These endolysin genes are also neighboured by holin genes in the phage genomes. However, *lysL* gene is located in an incomplete prophage PLl460-1, where it is unknown which parts of the prophage have been rearranged. Hence, the set and the order of the genes may have earlier been different in PLl460-1. Phages can evolve by recombinations, causing gene exchanges (Oechslin et al 2022). It is therefore possible that the origin of *lysl* gene is not prophage PLl460-1, or that even larger part of the prophage region is a result of recombinations and genomic rearrangements. Based on BLAST nucleotide sequence comparisons, identical (≥ 99%) prophage PLl460-1 fragment in the same location in the chromosome (Fig. 1), is found from several other *L. lactis* strains, for instance the strains SRCM103457, JXNPKM 1305, and D53 (accession numbers CP035757.1, JAKIVG010000004.1, and WKFC01000004.1). Thus, the mutations, rearrangements and possible recombinations in the prophage fragment causing gene exchanges have most likely happened earlier in an ancestor strain, and not in LAC460.

### Supplementary Information

Below is the link to the electronic supplementary material.

Supplementary file1 (DOCX 16 KB)
