# Supplementary material for: Analysis of the cell wall binding domain in bacteriocin-like lysin LysL from Lactococcus lactis LAC460
Source: Arch Microbiol. 2024 Jul 2;206(7):336. doi: 10.1007/s00203-024-04066-5 (PMC11219366; doi:10.1007/s00203-024-04066-5)
Supplement: Supplementary file 1 — Supplementary file1 (DOCX 16 KB) [file 203_2024_4066_MOESM1_ESM.docx]

**Supplementary Table 1.** PCR primers used in this study. Overlap extensions added to GFP R and LysL F primers are underlined.

| Primer name | Sequence (5’ 🡪 3’) | Use / Target | Reference/Source |
| --- | --- | --- | --- |
| LysL R | GGACCTCGAGTTATTTAAAATTAATATATTTTAATGG | *lysL* | This study |
| His-GFP F | ATGCACCATCACCATCACCACATGAGTAAAGGAGAAGAAC | *GFPuv* gene | This study |
| GFP R OE L | GTAATTCCTAAATGTTTGTAGAGCTCATCCATGCC | *GFPuv* gene, overlap extension to *lysL* | This study |
| LysL F OE GFP | GAGCTCTACAAACATTTAGGAATTACACAAAAAAACTGG | *lysL*, overlap extension to *GFP* | This study |
| pASG-IBA4 F | CTGCGTCACGGATCTCCACG | Vector pASG-IBA4 | Sorokina, 2015 |
| IBA noSS R | CATTTTTTGCCCTCGTTATCTAG | Vector pASG-IBA4 | Sorokina, 2015 |
| UC-CVfw | GTGCCTATGCTCCGTTAGTC | CWPS genotype A | Mahony et al., 2013 |
| UC-CVrv | CGAGGGCCAATCTCTTTACC | CWPS genotype A | Mahony et al., 2013 |
| IL-KFfw | GATTCAGTTGCACGGCCG | CWPS genotype B | Mahony et al., 2013 |
| IL-KFrv | AGTAAGGGGGCGGATTGTG | CWPS genotype B | Mahony et al., 2013 |
| MG-SKfw | AAAGCTCATCTTTCCCCTGTTGT | CWPS genotype C | Mahony et al., 2013 |
| MG-SKrv | GCACCATAGTCTGGAATAAGACC | CWPS genotype C | Mahony et al., 2013 |
| pA | AGAGTTTGATCCTGGCTCAG | CWPS control | Edwards et al., 1989 |
| pE’ | CCGTCAATTCCTTTGAGTTT | CWPS control | Edwards et al., 1989 |
